# A Single-Step Genome-Wide Association Study for Semen Traits of Egyptian Buffalo Bulls

**DOI:** 10.3390/ani13243758

**Published:** 2023-12-05

**Authors:** Ayman G. EL Nagar, Mohamed M. I. Salem, Amin M. S. Amin, Maher H. Khalil, Ayman F. Ashour, Mohammed M. Hegazy, Hamdy Abdel-Shafy

**Affiliations:** 1 Department of Animal Production, Faculty of Agriculture at Moshtohor, Benha University, Benha 13736, Egypt; maher.khalil@fagr.bu.edu.eg; 2 Department of Animal and Fish Production, Faculty of Agriculture (El-Shatby), Alexandria University, Alexandria 21545, Egypt; mmisalem@gmail.com; 3 Animal Production Research Institute, Agricultural Research Center, Dokki, Giza 12619, Egypt; amin_ama@yahoo.com (A.M.S.A.); hagar_ashoor@yahoo.com (A.F.A.); mmgad2120@yahoo.com (M.M.H.); 4 Department of Animal Production, Faculty of Agriculture, Cairo University, El-Gamma Street, Giza 12613, Egypt; hamdyabdelshafy@agr.cu.edu.eg

**Keywords:** semen traits, X-chromosome, candidate genes, genomic selection, ss-GWAS, buffalo

## Abstract

**Simple Summary:**

A study was conducted to investigate five semen traits in Egyptian buffalo bulls using a single-step genome-wide association study for genomic evaluation. The study aimed to identify associated genomic regions and potential candidate genes. The X-chromosome was the most crucial, accounting for 23.43% of the overall genomic variance observed in these semen traits. Numerous potential candidate genes associated with the examined semen traits were identified within the genomic regions under investigation. In addition, a total of five new candidate genes were discovered within the genomic area examined for semen traits in Egyptian buffalo bulls.

**Abstract:**

The present study aimed to contribute to the limited research on buffalo (*Bubalus bubalis*) semen traits by incorporating genomic data. A total of 8465 ejaculates were collected. The genotyping procedure was conducted using the Axiom^®^ Buffalo Genotyping 90 K array designed by the Affymetrix Expert Design Program. After conducting a quality assessment, we utilized 67,282 SNPs genotyped in 192 animals. We identified several genomic loci explaining high genetic variance by employing single-step genomic evaluation. The aforementioned regions were located on buffalo chromosomes no. 3, 4, 6, 7, 14, 16, 20, 22, and the X-chromosome. The X-chromosome exhibited substantial influence, accounting for 4.18, 4.59, 5.16, 5.19, and 4.31% of the genomic variance for ejaculate volume, mass motility, livability, abnormality, and concentration, respectively. In the examined genomic regions, we identified five novel candidate genes linked to male fertility and spermatogenesis, four in the X-chromosome and one in chromosome no. 16. Additional extensive research with larger sample sizes and datasets is imperative to validate these findings and evaluate their applicability for genomic selection.

## 1. Introduction

Water buffaloes (*Bubalus bubalis*) are vital dairy animals with significant economic and cultural value in several regions across the world, such as Asia, South America, and the Mediterranean countries, including Egypt. The water buffalo, specifically river buffalo subspecies, was domesticated and introduced to Egypt and Italy during the eighth century by the Arabs [1]. According to FAOSTAT, 2023 “https://www.fao.org/statistics/en (accessed on 15 June 2023)”, the Egyptian buffalo ranks fourth in global milk production and sixth in meat production, indicating its significant contribution to production efficiency. The rankings highlight the perceived efficiency and productivity of the Egyptian buffalo in the context of global agriculture. Moreover, compared to other livestock species, buffalo exhibit a remarkable ability to thrive in hot, humid, muddy, and marshy habitats, thereby enhancing the economic feasibility of buffalo farming [2]. However, the untapped potential of buffalo in Egypt remains largely unexplored due to the lack of national large-scale records and adequate pedigree information. In order to address this limitation, the genomic information must be incorporated into the breeding evaluation program, thereby enhancing milk production traits and bull performance could be achieved [3]. Unfortunately, this aspect of evaluation has not yet been explored in the context of genomic information in Egyptian buffalo. The enhancement of buffalo breeding can be effectively accomplished by utilizing bulls exhibiting exceptional reproductive performance. Furthermore, identification of the genetic markers for male fertility can provide significant advantages in assessing semen characteristics, particularly in the context of artificial insemination techniques. 

Genome-wide association study (GWAS) has recently gained significant progress, facilitating rapid advancements in high-throughput genotyping and molecular technologies. The widespread adoption of GWAS has resulted in its integration into breeding programs for various livestock species, including swine [4], chickens [5], buffalo [6,7], and dairy cattle [8]. GWAS plays a vital role in the identification of markers and genomic regions associated with several economically important quantitative traits. A significant advancement has been made by introducing a novel approach known as single-step genomic best linear unbiased prediction (ssGBLUP) [9]. This approach has a distinct benefit and enhanced precision relative to the ordinary GWAS in simultaneously estimating breeding values for genotyped and non-genotyped animals and integrating genomic, pedigree, and phenotypic data into a unified model. Consequently, this has the potential to improve the accuracy of predicting the genetic merit of the animal [10]. The GEBVs obtained from ssGBLUP are converted into marker effects and marker weights, which are subsequently utilized in an iterative process to update the ssGBLUP solutions.

In practice, the application of GWAS has been employed to determine potential genes that are linked to semen traits in cattle [11]. Nevertheless, there is a paucity of research on genomic studies assessed for semen traits in buffalo. Therefore, the objectives of the present study were (1) to implement a ssGWAS methodology for semen traits in the Egyptian buffalo bulls using the Axiom^®^ Buffalo Genotyping 90 K array and (2) to identify the genomic regions and the potential candidate genes associated with the studied semen traits. This study contributes to understanding the genetic/genomic architecture underlying the reproductive traits in buffalo bulls. Moreover, this study holds practical implications for buffalo breeders aiming to enhance the reproductive performance of their animals through selective breeding strategies. 

## 2. Materials and Methods

### 2.1. Ethical Statement

This study is part of a project entitled “A genomic approach to improve production and reproduction traits in Egyptian buffalo”, funded by the Science, Technology & Innovation Funding Authority (STDF), Egyptian Ministry for Scientific Research, Egypt. The Institutional Animal Care and Use Committee of the Agricultural Research Center (ARC-IACUC), Egypt, reviewed and granted approval for the project’s protocols and procedures. The study was conducted under the approval number ARC-APRI-50-23. 

### 2.2. Animals and Phenotypes

A total of 8465 semen ejaculates were collected throughout 14 consecutive years from 2009 to 2022 from 115 buffalo bulls. These bulls were reared in two different herds; 29 buffalo bulls (with 7626 ejaculates) originated in the International Livestock Management Training Center (IMTC) at Sakha, Kafr El-Sheikh Governorate, and 86 buffalo bulls (with 839 ejaculates) originated in Mahalet Mousa farm (MMF), Kafr El-Sheikh Governorate. The two farms belong to the Animal Production Research Institute (APRI), Ministry of Agriculture, Dokki, Egypt. Out of these 115 buffalo bulls with phenotypes, a total of 96 bulls have been genotyped. The original pedigree file involved 10,748 animals, with 179 sires and 2507 dams. The bulls of IMTC weighing 350–400 kg live body weight were fed a ration of 4 kg of concentrate feed, 3 kg of clover hay, and 4 kg of rice straw per day, and semen was collected twice a week at 8 a.m. from each bull. However, the bulls of MMF weighed 400–500 kg live body weight, fed concentrate feed contained 16% crude protein, rice straw, maize silage (during the summer season), and Egyptian Berseem, Trifolium alexandrinum (during the winter season). In that farm, every bull was fed twice a day, at 7 a.m. and 3 p.m., and semen was collected from each bull twice weekly (on Sunday and Wednesday). All the bulls used for semen collection were free of pathogenic diseases and healthy, with a likely body condition score of 3.5. These differences in the management followed in the two farms have been considered by adding the herd fixed effect in the model of analysis. In the two farms, the semen was collected through an artificial vagina (IMV Technologies, L’Aigle, France), maintained at 42–45 °C, and immediately transferred to the laboratory in a water bath (37 °C) for further semen evaluation. The semen traits examined were ejaculate volume (VOL), sperm mass motility % (MM), living sperm % (LS), abnormal sperm % (AS), and sperm concentration (CONC). The ejaculate volume was precisely measured in milliliters to the nearest 0.1 mL using a graduated glass tube. Semen mass motility (%) was calculated using the proportion of spermatozoa wave motion in a drop of semen placed on a glass slide. The live sperms (%) and abnormal sperms (%) were measured according to the methods of Hackett et al. (1965) [12] and Blom et al. (1983) [13]. The sperm concentration (10^9^ spermatozoa/mL) in each ejaculate was measured using a Neubauer hemocytometer.

### 2.3. Genotyping and Quality Control

Blood samples were collected from the jugular vein and transferred into 15 mL Falcon tubes containing 1 mL of 0.5 M EDTA as an anticoagulant. The tubes were immediately chilled on ice to preserve the samples. Genomic DNA was subsequently extracted from the whole blood samples using the QIAamp^®^ DNA Blood Mini Kit (QIAGEN, Hilden, Germany). The Axiom^®^ Buffalo Genotyping 90 K array was utilized for genotyping, which was conducted by the Laboratorio Genetica e Servizi LGS-AGROTIS, Cremona, Italy (Agrotis S.r.L.-LGS, Cremona, Italy “http://www.lgscr.it/ENG/index.html (accessed on 1 February 2023)”. The raw signal intensities (CEL files) of the Single Nucleotide Polymorphisms (SNPs) were processed and converted into genotype calls using Axiom Analysis Suite, version 5.2, which can be accessed at “https://www.thermofisher.com/order/catalog/product/550431 (accessed on 15 February 2023)”.

The initial genotypes comprised 123,040 SNP markers, evenly distributed across 24 autosomes and the X-chromosome (indicated as chromosome no. 25). The overall genotyping rate was 0.98. In order to ensure the quality of the data, a series of quality control steps were performed using PLINK 1.9 [14]. These procedures were performed involving the removal of SNPs with unknown and/or duplicate positions, low minor allele frequency (MAF < 0.01), or excessive missing genotypes per SNP (>0.10). Additionally, individuals with a low call rate (<0.10) were excluded from the analysis. Following the application of these filtering restrictions, the final dataset consisted of 192 genotyped animals (96 genotyped bulls and 96 genotyped cows) and 67,282 SNPs, with an improved genotyping rate of 0.99. The main objective of this study was to evaluate the semen quality in buffalo bulls. As a result, an additional 96 females were included in the genotyped dataset. This inclusion aimed to improve the accuracy of prediction during the construction of the H-matrix for single-step genomic evaluation.

### 2.4. Statistical Analyses 

The mixed model procedure implemented in the R program was utilized to evaluate the significance of all available fixed effects [15]. Following the test, only the factors that demonstrated statistical significance were chosen for inclusion in the genetic analysis. Furthermore, prior to commencing the genetic analysis, a comprehensive examination of the data distribution was conducted to ensure the suitability for the subsequent steps.

The ss-GWAS was employed to evaluate each trait since this analysis procedure involves integrating phenotypes, genotypes, and pedigree information in one step using the BLUPF90 software family [16]. The animals were initially renumbered sequentially using the RENUMF90 software, and the resulting input files were then employed in the BLUPF90, PREGSF90, and POSTGSF90 programs for the purpose of conducting a single-step analysis. The univariate animal model was utilized to analyze the vector of y, which represents the phenotypic observations for each semen trait. The following univariate animal model was applied:

y=Xb+Za+Wp+ε

In this model, the vector b comprises all the fixed effects, including the effects of the herd and the combined effect of the year and month of collection of semen samples. Additionally, linear regressions of the age of the bull at semen collection were incorporated into the model. The vectors of a and p are representative of random additive genetic and permanent environmental effects, respectively, whereas ε denotes the vector of random residuals. In order to examine the association between the observations denoted by the vector y and the different factors, namely, **X**, **Z**, and **W** incidence matrices were utilized. These three matrices are responsible for linking the observations of y to the fixed effects, random animal effects, and random permanent environmental effects, respectively. The analysis was conducted with assumptions of $\propto \;\sim \;N\;(0,\;H\sigma_{a}^{2})$, $p\;\sim \;N\;(0,\;I\sigma_{p}^{2})$, and $\varepsilon \;\sim \;N\;(0,\;I\sigma_{\varepsilon }^{2})$, where **H** is a combined matrix from **A** (pedigree relationship matrix) and **G** (genomic relationship matrix), **I** is an identity matrix; and **σ^2^_α_**, **σ^2^_p_**, and **σ^2^_ε_** are additive, permanent environmental and residual variances, respectively. The inverse of the **H** matrix (**H*^−^*^1^**) was constructed according to Aguilar et al. (2010) [10] as: $H^{-1}=A^{-1}+\left[\begin{array}{cc} 0 & 0\\ 0 & G^{-1}-A_{22}^{-1} \end{array}\right]$, where **G*^−^*^1^** is the inverse of the genomic relationship matrix and $A_{22}^{-1}$ is the inverse of numerator relationship matrix. The **G** matrix was constructed based on [17] as $G=\frac{{ZZ}^{’}}{2\sum_{j=1}^{m}p_{j}(1-p)}$, where **Z** represents the difference between **M** matrix (matrix of genotypes with columns indicating the markers and rows representing the animals) and **P** matrix (frequency matrix of the second allele p_j_, expressed as 2p_j_).

The SNP effects were estimated using the ssGBLUP framework proposed by Wang et al. (2012) [9]. The mathematical representation of the function is given in matrix form as follows: $\hat{u}={DZ}^{\prime }\;{\left[{ZDZ}^{\prime }\right]}^{-1}{â}_{g}$, where **û** is the vector of SNP effect; **D** is the diagonal matrix containing weighting factors for the SNP effects; **Z** is the genotype matrix, and **â_g_** is the vector of breeding values estimated for genotyped animals. To calculate the variance explained by each SNP, the formula $\sigma^{2}={û}^{2}\;2p(1-p)$ was used, where **û** corresponds to the SNP effect as described earlier, and *p* denotes the allele frequency of the SNP [18]. The percentage of genetic variance explained by a window segment of 30 adjacent SNPs was calculated as follows: $\frac{Var(a_{i})}{\sigma_{a}^{2}}\times 100\%$, where **a_i_** i represents the genetic value of the i-th region that consists of 30 adjacent SNP and $\sigma_{a}^{2}$ is the total genetic variance [19].

### 2.5. Candidate Genes Identification

In the current study, a sliding-window approach implemented in the ss-GWAS was used to assess the proportional contribution of genomic variance attributed to each genomic segment for the aforementioned semen traits. The top ten window segments (each spanning 30 consecutive SNPs) were selected, i.e., accounting for the highest proportion of the genomic variance to identify candidate genes for each semen trait. Furthermore, in order to comprehend the chromosomal impact, the percentages of variance explained by the windows belonging to each chromosome were aggregated. This approach provides valuable insights into the genomic architecture underpinning male fertility traits and contributes to pinpointing potential genomic regions of interest. The gene information was extracted from each window segment proximal for every trait and from the comprehensive database and library provided by Thermo Fisher Scientific “https://www.thermofisher.com/order/catalog/product/550431 (accessed on 23 March 2023)” to gather the relevant data. The genomic position of each SNP and the distances between markers and genes were determined using the latest reference assembly of the buffalo genome (UOA_WB_1: GCA_003121395.1).

### 2.6. Enrichment Analyses and Functional Annotation

The functional enrichment and metabolic pathways analysis were conducted using version 6.8 of the “Database for Annotation, Visualization, and Integrated Discovery” (DAVID) v 6.8 [20]. The parameters suggested by the authors were used in the computation for the functional analysis. In addition, the functions for each annotated gene were obtained independently by utilizing the database containing all annotated functions from Ensembl Rapid Release and DAVID.

## 3. Results 

### 3.1. Descriptive Statistics of Semen Traits in the Egyptian Buffalo Bulls

Table 1 shows the main statistical features of the five studied semen traits in the Egyptian buffalo bulls. The overall means for VOL, MM, LS, AS, and CONC were 3.78 mL, 62.15%, 61.07%, 4.47%, and 0.76 × 10^9^ spermatozoa/mL, respectively. The coefficient of variation (CV%) for semen traits exhibited a moderate range, spanning from 25.53% to 58.39%, as depicted in (Table 1). 

### 3.2. Genotype Filtering and Quality Control

Following the quality control steps, the dataset comprised 67,282 SNPs, reflecting a substantially improved genotyping rate of 0.99. For the raw and filtered number of SNPs, the initial number of SNPs ranged from 1779 to 8533, while the filtered number of SNPs ranged from 1152 to 5526 (Table 2). The raw and filtered minor allele frequencies were moderate and ranged from 0.24 to 0.32 for initial MAF with an average of 0.28 and ranged from 0.28 to 0.34 for filtered MFA with an average of 0.32. The gap size between adjacent SNPs was high and ranged from 35.59 to 51.51 kb with an average of 38.9 ± 28 kb (Table 2). The raw and filtered whole chromosomal lengths were 2617 and 2614 Mb, respectively, in the whole buffalo genome (Table 2).

### 3.3. ss-GWAS for Semen Traits in the Egyptian Buffalo Bulls

**Figure 1:**
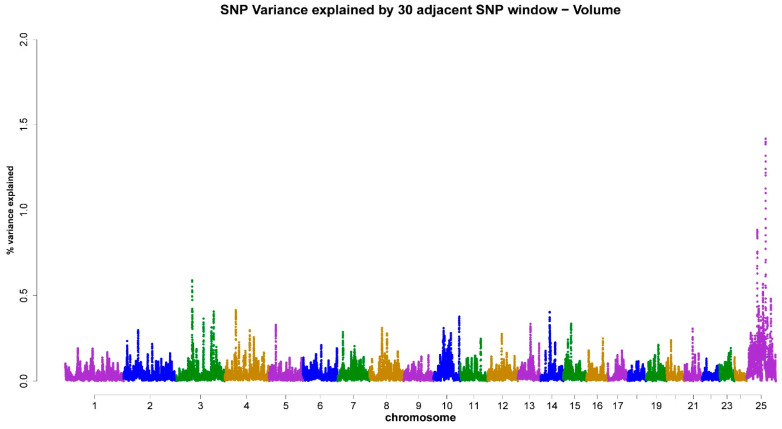
ssGWAS results of ejaculate volume (VOL) in the Egyptian buffalo bulls. Single nucleotide polymorphisms on different chromosomes are denoted by different colors. Each dot represents one SNP. The X-axis represents 25 chromosomes. The Y-axis represents the percentage of the explained variance by SNP.

**Figure 2:**
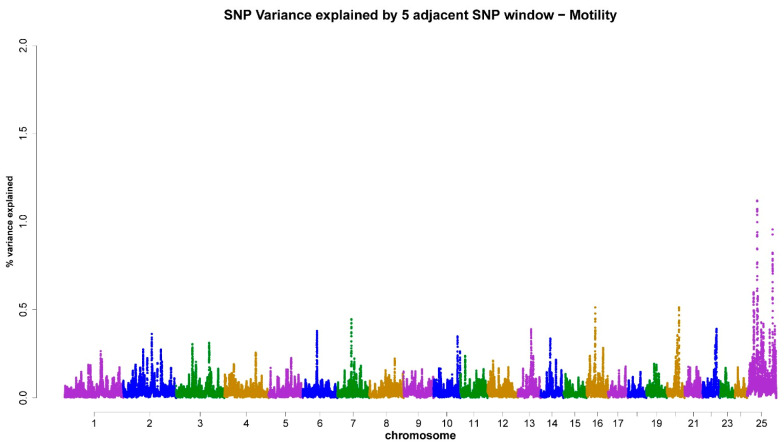
ssGWAS results of mass motility (MM) in the Egyptian buffalo bulls. Single nucleotide polymorphisms on different chromosomes are denoted by different colors. Each dot represents one SNP. The X-axis represents 25 chromosomes. The Y-axis represents the percentage of the explained variance by SNP.

**Figure 3:**
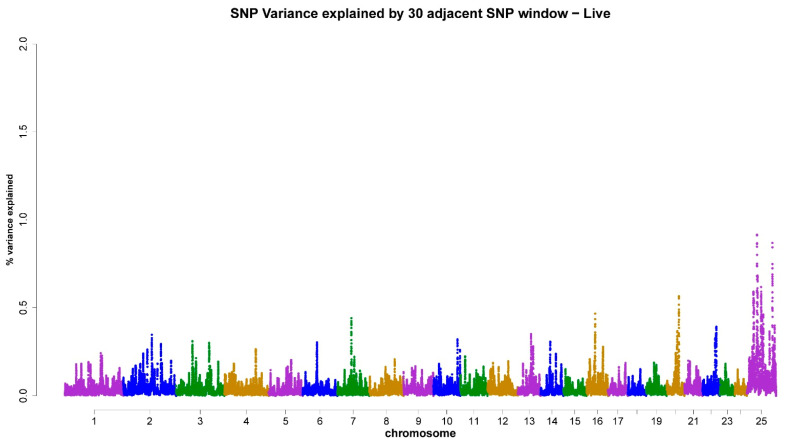
ssGWAS results of sperm livability (LS) in the Egyptian buffalo bulls. Single nucleotide polymorphisms on different chromosomes are denoted by different colors. Each dot represents one SNP. The X-axis represents 25 chromosomes. The Y-axis represents the percentage of the explained variance by SNP.

**Figure 4:**
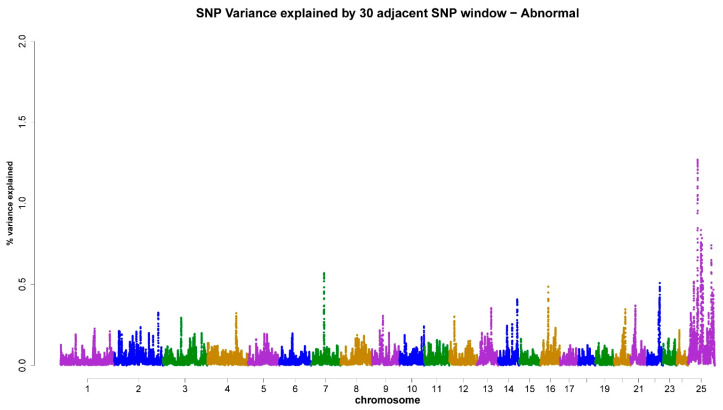
ssGWAS results of percentage of abnormal sperms (AS) in the Egyptian buffalo bulls. Single nucleotide polymorphisms on different chromosomes are denoted by different colors. Each dot represents one SNP. The X-axis represents 25 chromosomes. The Y-axis represents the percentage of the explained variance by SNP.

**Figure 5:**
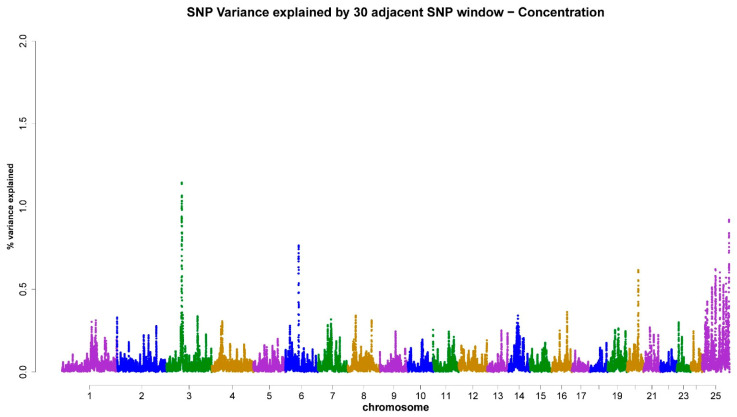
ssGWAS results of sperm concentration (CONC) in the Egyptian buffalo bulls. Single nucleotide polymorphisms on different chromosomes are denoted by different colors. Each dot represents one SNP. The X-axis represents 25 chromosomes. The Y-axis represents the percentage of the explained variance by SNP.

As shown in Table 3, the X-chromosome accounted for 4.18% (Figure 1), 4.59% (Figure 2), 5.16% (Figure 3), 5.19% (Figure 4), and 4.31% (Figure 5) of the genomic variance related to VOL, MM, LS, AS, and CONC traits, respectively. Collectively, across the five semen traits, the X-chromosome explained 23.43% of the genomic variance, contrasting with just 8.4% attributed to the joint effect of the remaining eight chromosomes (3, 4, 6, 7, 14, 16, 20, and 22). Accordingly, the X-chromosome was the most critical chromosome compared with other autosomes. Within the set of regions under investigation, it has been observed that two specific genomic regions, spanning a chromosome length of 69–77 Mb and 81–92 Mb, display associations with scrotal circumference. Additionally, another distinct region located at 40–55 Mb exhibits polymorphisms that are correlated with the percentage of normal sperm.

### 3.4. Functional Annotation and Identification of Putative Candidate Genes for Semen Traits

The genomic regions that have been identified are located within or close to a collective total of 50 genes. The annotated genes associated with the semen traits in the Egyptian buffalo are described in Table 4. Regarding these genes, 33 genes are situated in the X-chromosome, while the remaining 17 are placed in the other chromosomes (3, 4, 6, 7, 14, 16, 20, and 22). However, all the genes investigated here are considered protein-coding genes, except one pseudo-coding protein described as dynein light chain 1, cytoplasmic-like gene (*LOC102401551*, Table 4).

The novel annotated gene (*LOC102398394*) with the Ensembl gene ID ENSBBUG00015018582 within the genomic region length of 48.19 to 121.68 Mb associated with VOL and it is known as melanoma-associated antigen D2 (Table 3 and Table 4). The novel gene (*LOC112582129*) annotated by the Ensembl gene ID of ENSBBUG00015019729 is located on the X-chromosome within the genomic region length of 48.19 to 121.68 Mb, known as cancer/testis antigen 47A-like gene and associated with VOL trait (Table 3 and Table 4). The novel gene (*LOC102391626*), the Ensembl gene ID of ENSBBUG00015015043, is located in the X-chromosome within the genomic region length of 29.12 to 127.08 Mb and associated with MM, LS, AS, and CONC traits (Table 3). This gene is known as actin-related protein T1 (Table 4). The novel gene (*LOC102404920*) nominated as a sodium/hydrogen exchanger 2-like gene was identified on a genomic window of X-chromosome, and it is associated with LS, AS, and CONC traits in the Egyptian buffalo (Table 3 and Table 4). A novel gene (*LOC102397107*) with an Ensembl gene ID of ENSBBUG00015009483 has been observed (Table 4). This gene is located oon chromosome 16 at a position of 33.86 Mb. It has been found to be associated with MM, LS, and AS traits (Table 3). 

## 4. Discussion

The overall mean values for VOL, MM, LS, AS, and CONC exhibited a degree of similarity to those previously reported by Salem et al. (2023) [21] based on a subset of the current data from the IMTC herd. The authors reported that the measured values for VOL, MM, LS, AS, and CONC were 3.89 mL, 62.37%, 60.64%, 3.94%, and 0.67 × 10^9^ spermatozoa/mL, respectively. Additionally, the present results fall within the range found in earlier studies in dairy and buffalo bulls [22,23]. In the Egyptian buffalo bulls, Mahmoud et al. (2013) [24] revealed that the overall means for VOL, MM, and LS were 2.9 mL, 70.9%, and 65.8%, respectively. Khattab et al. (2015) [22] reported that the actual means for VOL, MM, and LS were 3.26 mL, 58.89%, and 65.96%, respectively. Similarly, the mean values for VOL, LS, AS, and CONC were reported by Gabr et al. (2018) [25] as 2.6 mL, 67.3%, 18.4%, and 526.28 × 106 sperm/mL, respectively. Additionally, Rushdi et al. (2017) [26] reported values of 66.20%, 70.58%, and 15.15% for MM, LS, and AS, respectively. In the Indian Murrah buffalo bulls, Kumar et al. (2023) [27] observed that the actual means of ejaculate volume and sperm concentration were 2.82 mL and 1040.12 million/mL, respectively. Variations in the mean values for semen traits observed in this study and those reported by different researchers working on different breeds of dairy or buffalo bulls may be explained by differences in genetic makeup, reproductive and health status of bulls, age of bulls, frequency of collection, teamwork in collection, nutrition, season and year of collection, and management [21]. These CV figures agreed with those published by Khattab et al. (2015) [22], who demonstrated that the CV% range for Egyptian buffalo bulls’ semen traits was 21.86% to 38.61%.

A comprehensive process of genotype filtering and quality control was conducted to ensure the robustness of the dataset. Approximately 45% of the raw genotypes were excluded from further analysis due to various reasons. The aforementioned factors include unknown or duplicated positions, instances of missing genotypes exceeding the threshold of 10% per SNP, and MAF falling below the established threshold of 0.01. This meticulous filtration process guarantees the reliability and integrity of the subsequent analyses conducted in the current study. The current MAF values were in accordance with those of El-Halawany et al. (2017) [6], who conducted GWAS analyses on the Egyptian buffalo, indicated divergent genotyped SNPs, stating an average gap spacing of 62.66 ± 67.10 and an MAF of 0.30 ± 0.13. The present filtered SNPs are consistent with those GWAS for milk production traits in the Egyptian buffalo reported by Abdel-Shafy et al. (2020) [7], who revealed an average distance between SNPs of 40.8 ± 32.0 kb and an average MAF of 0.29 ± 0.13 across chromosomes. This variance underscores the potential influence of diverse genetic backgrounds or experimental conditions on SNP distribution and MAF estimation.

The optimization of buffalo breeding practices can be significantly improved by selectively identifying buffalo that demonstrate superior reproductive performance. Consequently, the identification of genetic markers for male fertility using GWAS can offer substantial benefits in evaluating semen traits, including VOL, MM, LS, AS, and CONC, which would be particularly relevant with the increased application of artificial insemination. In the current study, a sliding-window approach implemented in the ss-GWAS was used to assess the proportional contribution of genomic variance attributed to each genomic segment for the aforementioned semen traits. We selected the top ten window segments (each spanning 30 consecutive SNPs) that account for the highest proportion of the genomic variance for each trait. Furthermore, in the pursuit of comprehending the chromosomal impact, the percentages of variance explained by the windows belonging to each chromosome were aggregated. This approach provides valuable insights into the genomic architecture underpinning male fertility traits and aids in pinpointing potential genomic regions of interest. 

Research on the area of dairy cattle breeding has emphasized the significance of the X-chromosome in relation to bull conception rates and semen-quality traits, including sperm concentration and motility [28,29]. Moreover, the X-chromosome relevance in exploring genes associated with spermatogenesis and male fertility has been proposed by Mueller et al. (2013) [30], highlighting the influence of the X-chromosome on genome-wide recombination and fertility. The investigation reported by Liu et al. (2019) [31] revealed that the enrichment of genes related to sexual development and reproduction is based on the sex chromosomes. Fortes et al. (2020) [32] evidenced a significant influence of the X-chromosome on bull fertility within two bovine populations. A study by Fortes et al. (2013) [33] has documented that the X-chromosome in bovines exhibits multiple regions linked to sperm morphology and testis development. To further this comprehension, a study conducted by Suchocki et al. (2015) [34] examined Polish Holstein-Friesian bulls and investigated semen characteristics using a GWAS. The researchers discovered a highly significant correlation between the X-chromosome and various semen traits, including sperm concentration, semen volume, motility score, and spermatozoa count. This finding indicates that the X-chromosome plays a crucial role in determining the quality of semen. In a similar context, Pacheco et al. (2020) [35] identified three candidate genes (*FAM9B*, *TBL1X*, and *PIH1D3*) within the X-specific genomic region that exhibited strong correlations with sire conception rate, testosterone concentration, spermatogenesis, and sperm motility, respectively, in dairy bulls.

The alignment in the genomic loci (29.35 to 127.09 Mb in the X-chromosome) highlights the similarity between the influences of the two traits (MM and LS). This consistency provides evidence for the substantial genetic correlation previously reported by Salem et al. (2023) [21] in the Egyptian buffalo bulls (0.99 ± 0.01), based on a subset of current data from the IMTC herd. A study by Khattab et al. (2015) [22] revealed a robust genetic correlation of 1.0 ± 0.03 between MM and LS traits, indicating that the enhancement of one trait could potentially lead to improvements in the other. The findings reported by Khattab et al. (2022) [23] for Friesian bulls gave a consistent genetic correlation of 0.64 ± 0.01 between MM and LS traits. According to the existing literature, no prior studies have investigated GWAS concerning semen traits in buffalo bulls. However, numerous genomic regions associated with semen characteristics have been identified in dairy and beef cattle through GWAS. In this regard, these genomic regions were previously identified in the Holstein Friesian bulls [36], in the US Jersey dairy bulls [29], in the Chinese Holstein bulls [28], in the Italian Holstein bulls [37], and in crossbred beef bulls [38]. 

The E3 ubiquitin-protein ligase SIAH1-like gene identified in the X-chromosome (48.19 to 121.68 Mb) plays a vital role in the spermatogenesis processes [39]. In the same genomic region, the novel annotated gene (known as melanoma-associated antigen D2) is considered one of the melanoma antigen proteins family (*MAGE*). Most *MAGE* genes are found in the X-chromosome and are expressed during the early stages of spermatogenesis [40]. Based on the tissue expression pattern, the *MAGE* gene can be divided into types I and II [41]. Type II MAGEs, on the other hand, have widespread expression in numerous tissues. According to several studies, *MAGE* genes are crucial for embryogenesis and the development of germ cells [41]. Moreover, in the X-chromosome, *TRO,* commonly known as Trophinin, was identified, and this gene could improve sperm motility [42]. 

*GRIA3* gene was identified in the X-chromosome (48.19 to 121.68 Mb), and the mutation of this gene led to an increased risk of 20–30% in decreasing the libido [43]. In the same genomic region, a novel gene was identified (cancer/testis antigen 47A-like gene). This gene exhibits distinctive and limited expression patterns during different stages of spermatogenesis, including cell cycle progression, meiosis, and spermiogenesis. *AR* gene (located in the X-chromosome) is a ligand-dependent transcription factor that modulates testosterone signaling and is essential for spermatogenesis and fertility [44]. The effects of androgens, which are necessary for the growth and maintenance of the male reproductive system, are mediated through the *AR* gene. Destructive mutations in *AR* can cause disorders ranging from minor deviations to complete failure of normal male phenotypic development [45]. The key cell regulators of androgen signaling androgen receptors are DNA-binding transcription factors primarily activated by testosterone and 5-dihydrotestosterone.

The *AR* candidate gene had significant effects on sperm quality traits (abnormal spermatozoa rate) in boars [46]. Sarakul et al. (2018) [47] found that the *COL4A6* gene was associated with semen volume, number of sperm, and sperm motility in the Thai multi-breed dairy population. *ZC4H2* gene located in the genomic region of 29.12 to 127.08 Mb in the X-chromosome was identified as an RNF220 interacting protein, a partner protein of RNF220 that stabilizes RNF220 [48]. In some cases, it serves as a mediator for the interaction between RNF220 and target proteins [49]. The *ZC3H12B* gene (located in the 29.12 to 127.08 Mb genomic region of the X-chromosome) is a negative regulator of macrophage activation and may play a role in inflammatory disorders and host immunity in the same genomic region. A novel annotated gene was identified, and this gene is known as actin-related protein T1. This gene encodes a protein related to the cytoskeletal protein beta-actin, and it presumably participates in the production of spermatids since it makes up a significant portion of the calyx in the perinuclear theca of the mammalian sperm heads “https://www.genecards.org/cgi-bin/carddisp.pl?gene=ACTRT1 (accessed on 24 April 2023)”. This gene is primarily found in the male germ cells of mammals, and several of them have been linked to spermatogenesis and fertility [50]. *AMMECR1* was found to be associated with distinct stages of spermatogenesis, namely early prophase I spermatocytes and mitotic spermatogonia. The *POU3F4* gene is located on the X-chromosome and is nominated as the POU class three homeobox four, and this *POU3F4* is considered a potential candidate gene associated with the other POU domain transcription factors that have been implicated in the maintenance of spermatogonial stem cells in Brahman bulls [51]. Moreover, the *POU3F4* gene has a vital role in bovine testicular development [33]. 

The novel annotated sodium/hydrogen exchanger 2-like gene was identified in the X-chromosome. The inhibition of acrosome production decreases its expression, ultimately causing male infertility. Interestingly, a distinct *PFKFB* activity was detected in the testes and a human testes-specific, suggesting that the unique metabolic requirements of sperm may necessitate a divergent *PFKFB* family member [52]. The *GPR174* gene nominated as G protein-coupled receptor 174 was previously reported to play an essential role in inducing the acrosome reaction of bull sperm [53]. The *DACH2* gene is a marker of immature Sertoli cells and may have a role in reproduction and development [54]. Regarding the X-chromosome, the *BCAP31* gene could be a significant marker for spermatogenesis and apoptosis and plays a pivotal role in the control of testis function in humans [55] and sheep [56].

*KIF2B* gene is located on the genomic region length of 58.52 to 137.06 Mb of chromosome number three; known as a meiosis-related gene, it has been discovered to be hypomethylated in the sperm but hypermethylated in the somatic cells. The hypomethylated *KIF2B* has a positive correlation with the fertility of bulls [57]. The *KIF2B* gene affects spindle bipolarity by causing microtubule depolymerization to alter mitotic dynamics during spermatogenesis [58]. In the same genomic region of chromosome number three, the *PTK2B* was identified. This gene belongs to the family of focal adhesion kinases and can be activated in response to outside stimuli that work through receptor tyrosine kinases [59]. This gene is crucial for modifying actin at sperm-binding sites and encourages sperm integration into the oocyte [60]. Yang et al. (2019) [61] reported that the *PTK2B* gene plays a vital role in regulating bovine mastitis. In the genomic region length of 60.92 to 62.73 Mb of chromosome number three, the *CA10* gene was identified, and it could have a crucial role in the interconversion of carbon dioxide, bicarbonate, and other physiological processes in many tissues [62]. *CA10* gene might regulate buffaloes’ fat metabolism and reproductive development [63]. Moreover, Carvalho et al. (2019) [64] reported that the *CA10* gene was jointly associated with other genes controlling hot carcass weight in Nelore cattle.

In chromosome number six, within the genomic region length of 51.58 to 52.47 Mb, the *BARHL2* gene (BarH-like homeobox 2) was identified. It is a transcriptional regulator and a member of the BarH family of homeodomain proteins and has been shown to affect cell fate specification, differentiation, survival, and migration [65]. The *KCTD8* gene (located in chromosome number seven within the genomic region length of 53.23 to 54.27 Mb) is associated with the milk fat percentage, as reported in GWAS in Indian buffaloes [63]. In the same genomic region of chromosome number seven, *YIPF* was identified. This gene could be suggested to play a significant role in the pathophysiology of varicocele. The potential correlation between the expression of *YIPF* and the testicular inflammatory response, as well as the fluctuation in TNF expression level induced by varicocele, has been suggested [66]. In the genomic region 34.04 to 35.39 Mb of chromosome 14, the *BMP2* gene was identified, and it is physiologically expressed in the testis and acts as proliferative signals upon Sertoli cells and spermatogonia [67]. 

The novel annotated *UBQLN3* gene (*ubiquilin-3*) was identified within the genomic region of 33.86 to 36.95 Mb. All ubiquitin-like proteins or ubiquilin-3 have a carboxy-terminal ubiquitin-associated (UBA) domain and an amino-terminal UBL domain. The testis is where this gene primarily expresses itself [68]. However, it has been proposed that this particular gene may have an impact on the progression of the cell cycle during the process of spermatogenesis and has been linked to male fertility [69,70].

## 5. Conclusions

The present study focuses on the implementation of single-step genome-wide association studies (ss-GWAS) to analyze semen traits in Egyptian buffalo bulls, representing the inaugural application of ss-GWAS in this particular population. The X-chromosome mainly explained 23.43% of the total genomic variance across the five semen traits. In contrast, the remaining eight chromosomes (3, 4, 6, 7, 14, 16, 20, and 22) accounted collectively for 8.40% of the total genomic variance. This finding suggests that semen traits exhibit polygenic inheritance patterns rather than simple ones. Several candidate genes associated with male fertility and spermatogenesis were identified in the X-chromosome. In practice, five novel candidate genes were detected across the whole genomic regions studied for semen traits in the Egyptian buffalo bulls (four novel genes in the X-chromosome and one novel gene in chromosome 16). A more precise buffalo genomic map is required to identify the candidate genes affecting semen traits, considering a larger sample size that may help to improve the power of candidate gene detection and to obtain more accuracy in the genomic breeding values. 

## Figures and Tables

**Table 1:** Descriptive statistics for semen traits in the Egyptian buffalo bulls.

Trait ^1^	Mean	SD	Min	Max	CV (%)
Ejaculate volume, mL	3.78	1.79	0.50	11.50	47.35
Mass motility, %	62.15	16.30	5.00	95.00	26.23
Livability, %	61.07	15.59	3.00	88.00	25.53
Abnormality, %	4.47	2.61	1.00	44.00	58.39
Concentration ^2^	0.76	0.35	0.20	3.75	46.05

^1^*:* Total number of records is 8465 ejaculates; ^2^: Sperm concentration (10^9^ sperm/mL).

**Table 2:** Summary statistics of genotyped SNPs in the Egyptian buffalo using Axiom Buffalo Genotyping Array.

Chr.	Number of SNPs		MAF		Chr. Length [Mb]		Gap Size [kb] ^#^		
	Initial	Filtered *	Initial	Filtered *	Initial	Filtered *	Mean	SD	Max
1	8533	5526	0.25	0.29	201.95	201.95	36.55	22.36	716.57
2	7786	4993	0.24	0.29	188.83	188.83	37.83	27.05	699.06
3	7192	4564	0.27	0.31	175.41	175.39	38.44	27.10	762.66
4	6499	4192	0.27	0.29	165.14	165.14	39.40	29.32	719.43
5	5027	3229	0.29	0.30	127.53	127.50	39.50	40.53	1721.08
6	5064	3264	0.29	0.29	120.32	120.24	36.85	23.83	477.34
7	4722	3027	0.29	0.30	117.10	117.10	38.70	23.74	418.96
8	5031	3244	0.28	0.29	119.61	119.50	36.85	22.03	512.54
9	4341	2825	0.27	0.30	110.10	109.97	38.94	28.94	689.09
10	3929	2539	0.28	0.29	104.07	104.07	41.01	24.94	414.49
11	4012	2572	0.27	0.29	102.20	102.05	39.69	27.67	609.86
12	4461	2880	0.27	0.28	106.40	106.37	36.95	19.29	180.88
13	3385	2154	0.28	0.29	90.40	90.22	41.90	45.88	1229.15
14	3342	2146	0.27	0.29	83.09	82.88	38.64	23.93	501.28
15	3428	2192	0.28	0.28	82.03	82.03	37.44	21.27	197.54
16	3185	2040	0.29	0.30	84.41	84.36	41.37	43.17	1139.89
17	2937	1873	0.28	0.29	73.12	72.68	38.82	24.00	398.63
18	2737	1728	0.29	0.30	65.76	65.69	38.04	32.20	560.14
19	2968	1940	0.28	0.30	71.58	71.54	36.90	18.94	235.90
20	2637	1691	0.30	0.31	68.47	68.45	40.50	28.75	386.39
21	2557	1703	0.28	0.29	60.72	60.58	35.59	18.07	231.64
22	2529	1633	0.28	0.30	61.97	61.69	37.80	20.60	173.44
23	2172	1410	0.32	0.34	51.44	51.18	36.32	21.94	388.82
24	1779	1152	0.30	0.34	42.13	42.00	36.49	22.36	264.13
X-chromosome	4891	2765	0.31	0.31	143.40	142.37	51.51	62.41	1427.38
UnPos	17,896	---	0.25	---	---	---	---	---	---
Overall	123,040	67,282	0.28	0.30	2617	2614	38.88	28.01	1721.08

Chr: chromosome; Mb: mega base; kb: kilo base; UnPos: un-positioned SNPs; MAF: minor allele frequency; * Number of SNPs after applying filtering restrictions (see materials and methods for further details) and duplicates SNP position; ^#^ Distance between adjacent SNPs after cleaning the data.

**Table 3:** The percentage of genomic variance explained by relevant genomic region ^1^ associated with semen traits in the Egyptian buffalo bulls.

Chromsome No.	Genomic Map Position (Mb)	Trait (Genomic Explained Variance %)	Gene ID (The Nearest Genes to the Position of the Chromosomal Window Segment)
X-chromosome	48.19–121.68	VOL (4.18%)	*LOC102414874*, *LOC102401551*, *LOC102398394*, *TRO*, *CHRDL1*, *GRIA3*, *LOC112582129*, *AR*, *OPHN1*, *COL4A6*
X-chromosome	29.35–127.08	MM (4.59%)	*ZC4H2*, *ZC3H12B*, *LOC102391626*, *SMARCA1*, *DMD*, *GNL3L*, *ITIH6*, *P2RY10*, *LOC102415058*, *RTL9*, *AMMECR1*
X-chromosome	29.12–127.08	LS (5.16%)	*ZC4H2*, *ZC3H12B*, *LOC102391626*, *SMARCA1*, *DMD*, *GNL3L*, *ITIH6*, *P2RY10*, *LOC102415058*, *RTL9*, *AMMECR1*, *POU3F4*, *LOC102404920*
X-chromosome	29.12–127.08	AS (5.19%)	*PFKFB1*, *TBX22*, *GPR174*, *DACH2*, *LOC112582031*, *LOC102391626*, *SMARCA1*, *POU3F4*, *LOC102404920*, *RTL9*, *AMMECR1*, *DMD*
X-chromosome	47.85–142.21	CONC (4.31%)	*BCAP31*, *P2RY10*, *LOC102415058*, *LOC102414874*, *LOC102401551*, *LOC102391626*, *SMARCA1*, *POU3F4*, *LOC102404920*, *LOC102394334*, *ZIC3*, *MAGEH1*, *FOXR2*
3	58.52–137.06	VOL (0.99%)	*KIF2B*, *LOC102390200*, *PTK2B*
3	60.92–62.73	CONC (1.14%)	*CA10*
4	43.42–44.85	VOL (0.42%)	*CSF2RB*
6	51.58–52.47	CONC (0.76%)	*ZNF326*, *BARHL2*
7	53.23–54.25	MM (0.45%)	*KCTD8*, *YIPF7*
7	53.26–54.27	AS (0.57%)	*KCTD8*, *YIPF7*
14	34.03–35.39	VOL (0.40%)	*FERMT1*, *BMP2*
16	33.86–36.95	MM (0.51%)	*LOC102402176*, *LOC102397107*
16	33.86–36.95	LS (0.47%)	*LOC102402176*, *LOC102397107*
16	33.86–36.95	AS (0.49%)	*LOC102402176*, *LOC102397107*
20	46.22–47.48	MM (0.51%)	*BLM*, *CRTC3*
20	46.22–47.48	LS (0.57%)	*BLM*, *CRTC3*
20	46.22–47.48	CONC (0.61%)	*BLM*, *CRTC3*
22	49.91–50.83	AS (0.51%)	*LOC102389067*, *CDH7*

^1^: The top 10 SNP-windows explained the highest genomic variance; VOL: ejaculate volume; MM: mass motility; LS: sperm livability; AS: percentage of abnormal sperms; CONC: sperm concentration.

**Table 4:** The function of candidate genes located in relevant genomic region ^1^ associated with semen traits in the Egyptian buffalo bulls.

Candidate Gene ^2^	Chromosome No.	Gene Function
*LOC102414874*	X-chromosome	E3 ubiquitin-protein ligase SIAH1-like
*LOC102401551*	X-chromosome	Dynein light chain 1, cytoplasmic-like
*LOC102398394*	X-chromosome	Melanoma-associated antigen D2
*TRO*	X-chromosome	Trophinin
*CHRDL1*	X-chromosome	Chordin like 1
*GRIA3*	X-chromosome	Glutamate ionotropic receptor AMPA type subunit 3
*LOC112582129*	X-chromosome	Cancer/testis antigen 47A-like
*AR*	X-chromosome	Androgen receptor
*OPHN1*	X-chromosome	Oligophrenin 1
*COL4A6*	X-chromosome	Collagen type IV alpha 6 chain
*ZC4H2*	X-chromosome	Zinc finger C4H2-type containing
*ZC3H12B*	X-chromosome	Zinc finger CCCH-type containing 12B
*LOC102391626*	X-chromosome	Actin-related protein T1
*SMARCA1*	X-chromosome	SWI/SNF related, matrix associated, actin dependent regulator of chromatin, subfamily a, member 1
*DMD*	X-chromosome	Dystrophin
*GNL3L*	X-chromosome	G protein nucleolar 3, like
*ITIH6*	X-chromosome	Inter-alpha-trypsin inhibitor heavy chain family member 6
*P2RY10*	X-chromosome	Putative P2Y purinoceptor 10
*LOC102415058*	X-chromosome	P2Y receptor family member 10
*RTL9*	X-chromosome	Retrotransposon Gag like 9
*AMMECR1*	X-chromosome	AMMECR nuclear protein 1
*POU3F4*	X-chromosome	POU class 3 homeobox 4
*LOC102404920*	X-chromosome	Sodium/hydrogen exchanger 2-like
*PFKFB1*	X-chromosome	6-phosphofructo-2-kinase/fructose-2,6-biphosphatase 1
*TBX22*	X-chromosome	T-box transcription factor 22
*GPR174*	X-chromosome	G protein-coupled receptor 174
*DACH2*	X-chromosome	Dachshund family transcription factor 2
*LOC112582031*	X-chromosome	Protein FAM209B-like
*BCAP31*	X-chromosome	B cell receptor associated protein 31
*LOC102394334*	X-chromosome	Putative MAGE domain-containing protein MAGEA13P
*ZIC3*	X-chromosome	Zic family member 3
*MAGEH1*	X-chromosome	MAGE family member H1
*FOXR2*	X-chromosome	Forkhead box R2
*KIF2B*	3	Kinesin family member 2B
*LOC102390200*	3	Peptidyl-prolyl cis-trans isomerase A-like
*PTK2B*	3	Protein tyrosine kinase 2 beta
*CA10*	3	Carbonic anhydrase 10
*CSF2RB*	4	Colony stimulating factor 2 receptor subunit beta
*ZNF326*	6	Zinc finger protein 326
*BARHL2*	6	BarH like homeobox 2
*KCTD8*	7	Potassium channel tetramerization domain containing 8
*YIPF7*	7	Yip1 domain family member 7
*FERMT1*	14	FERM domain containing kindlin 1
*BMP2*	14	Bone morphogenetic protein 2
*LOC102402176*	16	Ubiquilin-1 or Ubiquitin-like proteins
*LOC102397107*	16	Ubiquilin-3
*BLM*	20	BLM RecQ-like helicase
*CRTC3*	20	CREB-regulated transcription coactivator 3
*LOC102389067*	22	Serpin B8
*CDH7*	22	Cadherin 7

^1^: The top 10 SNP-windows explained the highest genomic variance; ^2^: All types of the candidate genes are known as protein-coding genes except one gene described as pseudo protein; dynein light chain 1, cytoplasmic-like; Source: NCBI “http://www.ncbi.nlm.nih.gov/gene (accessed on 24 April 2023)”.

## Data Availability

The datasets of this study are confidential.
